# Exit interviews from two randomised placebo-controlled phase 3 studies with caregivers of young children with autism spectrum disorder

**DOI:** 10.3389/frcha.2024.1236340

**Published:** 2024-06-05

**Authors:** Natalia Hawken, Bruno Falissard, Carl Choquet, Clement Francois, Jean Tardu, Ramona Schmid

**Affiliations:** ^1^ HTA Department, Creativ-Ceutical, Paris, France; ^2^ Epidemiology Department, CESP/INSERM U1018, Psychiatrie du Développement et Trajectoires, Paris, France; ^3^ IM Franchise Department, Les Laboratoires SERVIER, Global Value, Access & Pricing, Suresnes, France

**Keywords:** exit interviews, caregivers, autism spectrum disorder, bumetanide, qualitative research, patient experience, patient-reported outcomes

## Abstract

**Introduction:**

Autism spectrum disorder (ASD) is characterised by difficulty with social communication and restricted, repetitive patterns of behaviour. This study aimed to improve understanding of the ASD patient experience with the treatment (bumetanide) regarding the changes in core symptoms and to assess changes considered as meaningful. To achieve this, qualitative interviews were conducted with caregivers of patients in two phase 3 clinical trials (NCT03715153; NCT03715166) of a novel ASD treatment.

**Methods:**

Caregivers were invited to participate in one interview after completion of the pivotal phase 3 study; for those of them who continued treatment after study completion, a second interview was held 3 months after trial completion. The interviews were conducted by qualitative researchers and followed a semi-structured interview guide. The interviews focused on patients’ ASD symptoms and their impact on their daily life before enrolment, and on any symptom changes patients experienced during the trial.

**Results:**

Out of the 13 eligible patients’ caregivers, 11 were interviewed up to two times at clinical sites in the UK, Spain, and Italy. The caregivers reported impairments in a wide range of skills: deficits in communication and social interaction; restricted, repetitive patterns of behaviour, interests, or activities; and cognitive, emotional, and motor impairments. Compared to before the trial initiation, caregivers also reported improvements in the following domains: communication, interaction with others, cognition, aggression, emotions, repetitive movements, eating, and sleeping.

**Conclusion:**

The exit interviews provided a rich source of qualitative data, allowing a deeper understanding of caregivers’ and patients’ experience of the disease and allowing us to understand what constitutes a meaningful change. These data also helped identify important experiences that may inform the patient-reported outcome measurement strategy for future trials in ASD.

## 1 Introduction

Autism spectrum disorder (ASD) is a complex neurodevelopmental disorder characterised by impaired social communication and restricted and repetitive behaviours (core symptoms) (1). Although symptoms of ASD are neurologically based, they exist on a continuum of impairment and manifest as behavioural characteristics that present differently depending on age, language level, and cognitive abilities. First symptoms may be observed as early as 2 years of age (2) with boys being diagnosed four times more often than girls (3).

Currently, there is no US Food and Drug Administration (FDA) or European Medicine Agency (EMA) approved pharmacological treatment for the core symptoms of ASD. Therapies based on applied behavioural analysis (ABA), as well as speech and occupational therapy, are used in children with ASD to improve their development. To manage related comorbidities, for example, autism-related irritability or attention deficit hyperactivity disorder (ADHD), a wide array of drugs may be used, including antipsychotics (risperidone in children aged 5–16 years, aripiprazole in children aged 6–17 years), antidepressants (selective serotonin reuptake inhibitors, tricyclics), stimulants, anticonvulsants, and anti-anxiety medication (4). However, risperidone’s beneficial effect on aggression is not accompanied by a reduction in core ASD symptoms and both risperidone and aripiprazole have important side effects, including sedation, vomiting, extrapyramidal syndromes, increased appetite, drowsiness, drooling, and body weight gain.

Two phase 3, double-blind, placebo-controlled clinical studies assessed the efficacy and safety of an sodium-potassium-chloride cotransporter 1 (NKCC1) antagonist, bumetanide, 0.5 mg twice daily in children with ASD [1 for children from 2 to less than 7 years old, a second one for children and adolescents from 7 to less than 18 years old (ClinicalTrials.gov identifier: NCT03715153; NCT03715166)] (5). Patients enrolled in the two clinical studies were offered the possibility of participating in a Compassionate Use Programme (CUP) after study completion or offered to pursue the study by entering into a 6-month extension period treated by bumetanide in countries where the local legislation does not accept the compassionate use approach. The change in Childhood Autism Rating Scale 2nd edition (CARS-2) total raw score after 6 months of treatment was the primary efficacy endpoint in both studies. Results of the Clinical Global Impressions Scale-Improvement (CGI-I) and Social Responsiveness Scale 2nd edition (SRS) were also collected.

Regulatory and research communities have endorsed qualitative “exit” interviews as a means of promoting patient-centric evaluation of treatment benefits. Indeed, data from such interviews can be used to supplement data collected via clinical outcome assessment (COA) measures implemented in clinical studies. While COA measures included in the trial provided a standardised means of evaluating and monitoring the efficacy and safety of products, conducting qualitative interviews as part of clinical research studies provides unique opportunities for in-depth exploration of the patient’s experience, especially in diseases like autism where the subtleties of patients’ experiences may not be fully captured by existing instruments. The assessment of treatment benefits in clinical trials often relies on the participants’ own descriptions (i.e., self-report) of their experiences and emotions (6). However, in mental disorders, and ASD in particular, because of the patient’s difficulties in communication, abstract thought, and interpretation of emotions, assessments have to rely on an observer rating. Caregivers of children with ASD serve as the best proxy for their children’s experience.

Exit interviews in a subset of caregivers of patients participating in the two phase 3 trials described above were conducted to understand the impact of autism before treatment and describe the impact of bumetanide on the core symptoms in autistic children and adolescents. More specifically, they were aimed at obtaining a better understanding of the experience with the changes in core symptoms, understanding changes that are meaningful to the patient and the caregivers, identifying unexpected treatment insights, and identifying any potential unmet needs.

## 2 Materials and methods

### 2.1 Ethical considerations

This interview study was conducted independently of the clinical study procedures. The study was conducted in accordance with the Declaration of Helsinki and was approved by the national ethics board (UK) or local ethics boards from the respective sites.

### 2.2 Site selection

With no guarantees regarding patient participation and the investment associated with study set-up and initiation (including ethical approval and translation of study materials), the sites with the greatest proportion of eligible patients were targeted for recruitment. Each pivotal phase 3 clinical study enrolled 211 participants (of which 185 completed study NCT03715153). The objective was to enrol a subset of 30 participants in the interview study. Based on the number of patients still enrolled in the pivotal phase 3 clinical studies and the time to obtain Ethics Committee approval, it was estimated that a maximum of 15 participants could enrol in the interview study.

### 2.3 Recruitment

Caregivers of patients who had recently completed participation (within the past month) in one of the two pivotal phase 3 clinical studies were invited to take part in this interview study at clinical sites in the UK, Spain, and Italy and to take part in up to two interviews: one after completion of the pivotal phase 3 clinical study; and for those of them who continued treatment after study completion, a second interview was held after 3 months of treatment.

Participants contacted by the study centre who expressed interest and signed the informed consent were contacted to schedule a telephone interview. The informed consent includes the right to access limited information from the phase 3 clinical studies. Informed consent was collected electronically in Spain and Italy and in person in the UK. Only the primary caregiver (defined by the parents themselves) was interviewed (no joint interview to avoid influence/interactions).

In September 2021, the results of the clinical studies were released. Due to the potential bias linked to data availability, it has been decided to end the recruitment at that stage. In total, 11 parents were interviewed.

### 2.4 Individual interviews

Semi-structured concept elicitation interviews lasting approximately 60 min were conducted via telephone by trained qualitative researchers in the local language. The content of the interview guide was reviewed by a clinical expert and two patient advocates (parents of patients with ASD) and was translated from English into the local language (where applicable).

Initial discussions with participants were broad and open-ended to establish rapport and facilitate spontaneous elicitation of ASD symptoms and concepts relevant to their experience with the clinical studies. Questions were designed to provide insight into participants’ evaluation of their child’s symptoms before and after enrolment in the phase 3 clinical study. After an icebreaker, participants were asked to describe the symptoms their children presented before their enrolment in the phase 3 clinical study. In the second part, participants were asked whether they observed any changes in their child’s symptoms after the start of the phase 3 clinical study.

Participants were compensated (€20 voucher) for the time taken to complete the interview once it was accepted by the ethics committee. All interviews were audio-recorded, transcribed verbatim, and anonymised.

All interviews were conducted before unblinding of the study. Neither the interviewer nor the participants knew to which treatment arm they were assigned at the time that the interviews were conducted.

### 2.5 Analyses qualitative data

Interview transcripts were imported into Atlas.Ti, a software package used to facilitate the storage, coding, and analysis of qualitative data. The thematic analysis (7) process involved creating and assigning data-driven codes related to the research aim. The analysis consisted of the attribution of tags (a code made of one to two words) summarising the idea being conveyed to a word, a sentence, or a paragraph of each of the transcripts. Each transcript was coded individually. As new codes emerged, transcripts were re-read and analysed to ensure all codes were consistently applied. These codes were grouped according to underlying themes.

#### 2.5.1 Explorative relationship between caregivers’ experience and clinical data

As clinical data were available for the patients in our sample, an explorative comparison was done to provide some potential insights into the relationship between the clinical data and patient/caregiver experience. Patient improvement was categorised by the coder as no improvement, minimal improvement, improvement, or major improvement, based on feedback received during the second part of the interview.

### 2.6 Sociodemographic and clinical data

#### 2.6.1 Sociodemographic data

Participants completed a brief questionnaire on sociodemographic and general health characteristics. This questionnaire included items on gender, age, education, employment status, and marital status of the caregiver and gender, age, education, and general health conditions of the patient. Descriptive statistics (e.g., mean, frequency) were used to summarise sociodemographic and other participants’ characteristics (Tables 1, 2).

**Table 1:** Demographics of respondents.

Demographic	Categories	Count/frequency/mean
Age (mean)		44
Gender	Female	10
	Male	1
Relation to patient	Mother	10
	Father	1
Employment status	Employed, full-time	5
	Employed, part-time	2
	Unemployed	1
	Homemaker	3
Highest level of education	Secondary/high school	3
	College degree	1
	Postgraduate degree	5
	Other	2
Marital status	Married/living with significant other	9
	Divorced/separated	2
Number of children	One	3
	Two	6
	Three	2
Ordinal place of child in family	First	7
	Second	3
	No answer	1
Other children with ASD	Yes	1
	No	10
Overall health	Excellent	3
	Very Good	1
	Good	5
	Fair	1
	No answer	1

**Table 2:** Demographics of patients.

Group	Categories	Count/frequency/mean
Treatment arm clinical study	Treatment	4
	Placebo	7
Gender	Male	9
	Female	2
Age, mean (range)		9.5 (6–14)

#### 2.6.2 Clinical data

Selected clinical data collected during the clinical study were used to describe the sample (e.g., study arm, diagnosis, and severity) at the end of the interview study. Data from three COAs collected in the clinical study were used to explore whether changes in their children’s symptoms, as described by the parents, were also captured by the measures included in the clinical study:

• CARS-2

One widely used rating scale for the detection and diagnosis of autism is the CARS (8, 9). The CARS-2 consists of a clinician-rated 15-item scale. There are two versions: one for children aged below 6 years and/or with an IQ < 80 (standard version); and the other for children aged 6 years or above with fluent language and without an intellectual disability (high-functioning version). Patients were included in the study if they had a score greater than or equal to 34, reflecting moderate to severe autism.

• SRS

The SRS (10, 11) is a quantitative scale that measures the presence and extent of autistic social impairment. The SRS measures reciprocal social behaviour and general behavioural problems that may underlie language difficulties and stereotyped behaviours common in autism (10–12) and is rated by the parents/caregivers of patients. Patients were included in the study if they had a score greater than or equal to 66.

• CGI-I

The CGI-I (13), which is used as a clinician-rated outcome measure in clinical trials for several psychiatric disorders, employs a 7-point scale to determine the individual’s improvement in response to treatment.

## 3 Results

### 3.1 Sample characteristics

Out of 13 eligible patients, 11 took part in the study and their caregiver was interviewed. Two caregivers of patients in the UK sample were not able to participate as they were not able to provide informed consent in person. All but one participant were the mothers of the respondents. Most of the patients included in the present study were boys. Out of the 11 patients included, 7 were in the placebo group in the clinical trials. All patients contacted agreed to participate. However, two patients from UK, who initially agreed, could not be included because they were unable to provide informed consent face-to-face. A summary of demographics is presented in Tables 1, 2.

### 3.2 Symptoms before study participation

Participants were asked to describe which ASD symptoms their children presented before enrolment in the phase 3 clinical study. Caregivers of patients reported impairments in a wide range of skills: deficits in communication and social interaction; restricted, repetitive patterns of behaviour, interests, or activities; and cognitive, emotional, and motor impairments. Based on the patients’ interviews, the descriptions of symptoms before enrolment in the clinical study have been presented in Table 3 and categorised as domains and sub-domains. Domains and sub-domains were not predefined but emerged from the data analysis. The results highlight that the sample consisted of children with heterogeneous profiles, ranging from children with a milder impairment in their expressive and receptive language abilities following a normal curriculum at school to children with a severe impairment in their expressive and receptive language abilities. The symptoms indicated by caregivers as being the most bothersome are summarised in Table 4.

**Table 3:** Reported symptoms before the clinical study.

Domain	Sub-domain	First-order code
Communication	Expressive language	No speech
		Echolalic speech
		Some words
	Understanding abstract communications skills	Doesn’t understand figures of speech/jokes
	Non-verbal communication	Doesn’t react to non-verbal communication
		Communicates with drawing/writing
	Making contact with a child	No contact
	Making eye contact	No eye contact
	Understanding what is expected of him/her	No understanding
Interactions with others	Engaging in activities	
	Showing interest in other children	No interest in other children
	Playing with others	No playing
		Plays next to other children
	Showing affection	Doesn’t show affection (a lot)
	Having friends	Doesn’t have friends
Play	Using toys in an unusual way	
	Plays alone	
Emotions	Anger	
	Tantrums	
	Fears/anxiety	Scared of loud noises
	Mood swings	
Aggression	Hitting others	
	Hitting him/herself	
Inflexibility of adherence with routines		
Cognition	Focus	Cannot focus for a longer period of time
	Maturity	Lack of maturity
	Hyperactivity	
	Being absent	
Repetitive behaviours		Flapping hands
		Lies on the floor
		Starts running without reason
		Repeats songs
		Rocking
		Jumping
		Talking to him/herself
Restricted interest		
Motor symptoms		Walking
		Gripping
		Writing/drawing
		Tripping/falling
		Tying shoes
Eating		Accept limited/specific food
		Doesn’t ask for food even if hungry
		Age inappropriate need to be fed
Sleep		Wakes up very early

**Table 4:** Top three most bothersome symptoms before enrolment in the clinical studies.

Domains		Count/frequency
Need for structures/routines		3
Communication	Expressive communication	5
Eating problem		2
Social skills	Lack of social relationship	3
Emotion	Fears/anxiety	4
	Mood swings	1
Repetitive movements		3
Aggression	Hitting others	3
	Anger/tantrums/frustration	5
Cognition	Problems in concentration for a longer period of time	1
	Hyperactivity	3

#### 3.2.1 Communication skills

Impairments in expressive communication skills were described in 7 of the 11 children before enrolment in the clinical study. Speech impairments range from no speech at all to the ability to speak some words or sentences without being able to hold a conversation and were rated as one of the three most bothersome symptoms by five caregivers. Of the 11 patients, 3 were described by their caregivers as unable to speak and unable to understand what was expected of them. Four other patients were described as being able to say some words or having echolalic speech.

*UK001: […] My son has profound autism, and he is non-verbal as well. So, it’s just absolutely knackered [laughs] is pretty much how we feel. More so at the beginning as well, so yeah*.

*ES004: OK. Then, he went to school and he started saying very punctual words, right? Very, very, just one thing and that was it, right? And like this, and the day, and until, well, before the study, nothing, um, he doesn’t communicate. Just when he asks for something, he says it, but he’s incapable of maintaining a conversation, with anyone*.

#### 3.2.2 Interaction with others

Severely impaired social interaction skills were described in 8 of the 11 children before study enrolment. Impairments were described as having no friends, not wanting to engage in social activities, and showing no interest in others. Impaired social skills were described by parents as a severe aspect of autism. However, despite the severity of this symptom, only three parents considered it one of the most bothersome symptoms.

*ES003: […] She doesn’t, she doesn’t have friends. I mean, children are afraid of her because she hits them. I mean, they don’t invite her to parties. […] she is not capable of being in class with the other children, she has to be in the special education classroom*.

*ES004: No, no, no, he wants to avoid, he wants to avoid conversation. The relationship he has with other people, so, he always avoids, or is not there, if I’m talking to someone, he doesn’t interrupt, but he doesn’t give his opinion either, he’s there, on the side, quiet*.

#### 3.2.3 Aggression

Aggression towards themselves or towards others was described by 5 of the 11 patients. Four of these patients were described as hitting others when frustrated. Two of the patients were hitting themselves when unable to control their emotions. However, despite the severity of this symptom, only four parents considered it one of the most bothersome symptoms.

*IT302: His aggressiveness, intolerance to frustrations and intolerance to “no.”*

*UK001: […] He’s very, very physical, […] So sometimes he will laugh and sometimes he’ll cry, so he will stick his fingers in your eye, and he grabs your neck. He will pull you down […] Like so when he goes for the eyes, he just keeps going and going and going and going, you tell him “no, no, no, no, no, no,” and try and stop him and then he’ll come behind you and he’ll sneak, and he’ll do it and he’s just… he’ll just have to do it*.

*IT302: A bad day is when X is very nervous, he cannot stand still for a second, he cannot sit, he cannot follow his lessons at school, he cannot do his homework. He realizes he cannot do what he has to do and he becomes frustrated and he becomes aggressive with himself*.

#### 3.2.4 Repetitive movements/stereotypies

Repetitive movements/stereotypies were described by 8 out of 11 parents and varied greatly in type from flapping hands to running or jumping. Stereotypies/repetitive movements were seen as a way for the children to regulate themselves.

*UK003: […] funny, making jokes, so he probably knows all the Chuck Norris jokes in the world, uh… ‘cause that’s what he’s into recently. So, I thought there is still some stuff which we’re struggling, so before the research uh… the main things which we’re struggling is the… is jumping and rocking, uh… which is like the way he self-regulates*.

*ES004: He lies on the floor, and that’s it. He sometimes starts running for no reason, it’s not the moment to run, or it’s not the moment to lie on the floor*.

*ES006: Oh, yes, yes, yes, he makes repetitive movements, like a lot of gestures with his hands, and that, yes, yes, he makes a lot of gestures, like a lot of movements with, with his body*.

Only one parent considered this as one of the least bothersome symptoms. The other parents described it as one of the most bothersome symptoms, due to the age-inappropriate behaviour.

*UK003: Uh, well definitely just jumping and rocking*.

#### 3.2.5 Cognition: ability to concentrate

Out of 11 patients, 5 were described as having problems concentrating for a longer period.

*IT302: The fact that he has to move continuously and he cannot stand still, causes him to be less concentrated on what he has to do.*

*UK001: […] He’s also got ADHD, so he’s just climbing around and not listening and not able to concentrate and just not really connecting with anyone, if that makes sense?*

#### 3.2.6 Emotions

Three different emotions were described as problematic for 8 out of 11 patients: anger, fear/anxiety, and mood swings. The parents of four patients described fear/anxiety as one of the most bothersome symptoms.

*ES004: He’s afraid, yes. He has fears, and there are things which cause him a lot of anxiety, right? But those are not the fears we sometimes have, that something is going to happen to us in the street, right? […] He’s very fearful in general*.

*ES006: So, […] getting angry, mood swings, um, also strong noises, echolalia, um, the restricted and repetitive interests, half obsessive*.

*UK002: He used to scare. He used to put both hands in the ear, he used to run to his room and hide there in the corner. I, I had to take him in the room and I had to shut the door and stay with him until all the pressure cooker, cooking is finished. He used to scare there and…*

#### 3.2.7 Need for structures/routines

Out of 11 parents, 7 described the need for routines and structures and the difficulty to deal with a change in a routine or a plan. Three parents described this symptom as one of the most bothersome one.

*IT001: She always has issues in accepting changes, because she doesn’t know what she might be facing. She wants explanations; we have to tell her what we are changing so that she can get ready*.

*IT302: The fact that he couldn’t accept change, changing a plan, or a sudden event. He can hardly accept it. We have to work on it, so that he accepts it*.

#### 3.2.8 Motor symptoms

Out of 11 parents, 9 reported that their child had one or more impairments in their motor skills.

*UK001: So, the hands on the ears and he will just be like, seems like he’ll shake his hands and… but he doesn’t walk right. He is hypermobile so that could cause for the funny… Like he leans on things. He’s very, um, like leans on everything as he walks, if that makes sense? Um…*

*IT302: Yes… well, let’s say that before the study he couldn’t tie his shoes*

*IT001: She had some issues in doing fine movements, she cannot be precise, but for example in drawing, she is fine. She cannot draw precise things; she has some issues with technical drawing*.

#### 3.2.9 Sleeping

Only two parents reported sleeping issues with their children. Parents described that their children were waking up very early in the morning or waking up in the middle of the night. Although one of the parents who reported sleeping issues reported it as having a major impact on their own quality of life, they did not identify their child’s sleeping issues as one of the most bothersome symptoms.

*UK002: Okay. Before, he used to wake up every two, three times a week in middle of the night. […] At least. But now, he sleeps like twice in two weeks’ time, he wakes up. There’s sometimes he doesn’t wake up, but he do wake up but not that much*.

*UK001: […], the main problem that we have is lack of sleep, uh, just complete and utter exhaustion just because we’re so tired, because he doesn’t sleep and also because it’s very draining*.

#### 3.2.10 Eating

Although ASD often comes with hypersensitivity to textures, eating problems were not commonly reported. Only three parents described that their children were not accepting all food textures and two parents described that they still needed to feed their children at an age that children would normally feed themselves.

*UK001: […] He used to just eat, um, Coco Pops, apples, and waffles and it had to be a normal waffle, but now he’s eating fish fingers. He is now eating burgers. He did eat a little bit of sausage before, but now he’s burgers. He’s eating pizzas. I know this sounds like rubbish food, but we were eating… He was eating nothing, um, before […]*

### 3.3 Symptoms during study participation

Parents reported changes in symptoms in the following domains: communication, interaction with others, cognition, aggression, emotions, repetitive movements, eating, and sleeping. Of the four patients on the active treatment, one did not report any improvement while the other three patients reported minimal improvement. Interestingly, only one patient from the placebo group reported no improvement while the other six reported minimal to major improvements.

#### 3.3.1 Improvement in communication skills

Of the three parents who described their children as being able to speak and unable to understand what was expected of them, only one of these parents described their child as being able to make more sentences.

*ES006: Make these noises, this has decreased a lot, when he was on the medication, and then he was more capable of making sentences and phrases. It was, it was important, I mean, he was able, in some way, to communicate better. […] These were the 2 more important things, and well, at school, they told me he was able to be much more quiet, paying attention, and relaxed in class*.

Of the four parents who described their children as being able to say some words or having echolalic speech, only one described an improvement in receptive communication. This improvement was rated as a major improvement for this parent.

*UK001: […] The first thing we, we noticed was, was the comprehension and that’s not just me, […] even his outreach people can just see that he’s just understanding more, like “[patient’s name] we’re going swimming,” he would go and get his swimming kit out from under the stairs, you know, um… Just, the, the comprehension was just… I don’t know if it just calmed him down and he was able to understand what we were saying, was the biggest thing. It was brilliant. […] Because he’s understanding, his comprehension, he’s not… It’s like he’s in our world now, whereas before, he was in [patient’s name]’s world, but now he’s so much more in this world, in this present*.

Another caregiver described that, although the child was still not able to talk, he was attempting to communicate his needs more.

*UK002: At least he do try to tell us where is things or he wants… you know. He, if he like, for example, if he want to sleep, he pull my hand and take me upstairs*.

#### 3.3.2 Interaction with others

Two parents described an improvement in their child’s interaction with others, and the improvement was described as moderate.

*IT001: Yes. If we attend a party, obviously, we haven’t been to parties lately, but I noticed that [patient’s name] in a situation in which I was not expecting her to react in that way, instead, she was very active, she introduced herself, even with people she had never seen before. […] The approach with the others, the initiative, she did not have it before*.

*IT302: I can say that [patient’s name] has increased his level of attention and also his ability to socialise was improved. When he was taking the drug, in the afternoon he really wanted to video call some friends to do his homework together. Right now, he is no longer taking the drug and he no longer feels the need to do it*.

#### 3.3.3 Aggression

Aggression towards themselves or towards others, or anger/tantrums, was described in 5 of the 11 patients. Four of these five patients were described as hitting others when frustrated. All five of the parents described aggression/anger as one of the most bothersome symptoms.

Four out of five patients were described as improving on aggression/anger issues.

*UK001: It [his aggressive behaviours] was a lot less, it was a lot less, not totally gone. We still have the issue of sleep, um, that triggers it a lot. […] So, if he’s had no sleep and he’s so tired and frustrated, then it, it will still raise its ugly head, but nothing like it was before*.

*ES003: Well, that she is able to sit on the chair and do an activity without getting angry. [laughs] I think they don’t put her in the classroom yet, or half an hour or so, that she is not capable of being in class with the other children, she has to be in the special education classroom. So, that, but well, that she no longer hits the teachers*.

*ES005: I mean, the behaviours had improved, he had, for example, instead of hitting, he sometimes hits, I’m not saying he doesn’t, it hasn’t disappeared completely, but it has almost disappeared. Instead of hitting, he has other strategies, like, for example, asking the teacher for help, or try to avoid those children and go somewhere on his own for 5 minutes to relax*.

#### 3.3.4 Cognition

Although 5 out of 11 patients were described as having problems concentrating for a longer period of time, 6 out of 11 caregivers noticed an improvement in their child’s ability to concentrate for longer periods of time.

*IT302: His level of attention. His attention was better compared to when he was not taking the drug. He could do his homework; he keeps his concentration for a longer time*.

*IT303: Hyperactivity as well, he was very calm, for example, if we would go out, and I would speak with a friend, he would remain calm. This is what I noticed, he would wait for me sitting down, for 10–15 minutes, very calm, he would wait for me to finish. Before the drug, he wouldn’t be like that. I couldn’t even think of stopping and having a chat with a friend. I had to keep him busy. Instead, (during the study) he would even listen to a conversation with a person he wouldn’t know. When we would go out together, he would be extremely calm. I would tell him “[patient’s name], wait” and he would wait calmly, he wouldn’t run around. I would tell him “[patient’s name], can you carry this?” … aspects that after the drug, he no longer does, just like before the drug*.

#### 3.3.5 Emotions

Only one parent reported an improvement in fear/anxiety during the study.

*UK002: He’s not scared of the noise—that’s a major improvement, much… uh, you know, he’s still watch in anxious way, but he doesn’t scare*.

Four of the five parents described an improvement in their child’s aggression/anger issues during the clinical study.

*UK001: It was a lot less, it was a lot less, not totally gone. We still have the issue of sleep, um, that triggers it a lot. […] So, if he’s had no sleep and he’s so tired and frustrated, then it, it will still raise its ugly head, but nothing like it was before*.

*ES005: So, if I have to tell you how he is right now, then, we have gone back. If I have to tell you how he was until he finished the treatment, so, overall, everything was much better. I mean, the behaviours had improved, he had, for example, instead of hitting, he sometimes hits, I’m not saying he doesn’t, it hasn’t disappeared completely, but it has almost disappeared. Instead of hitting, he has other strategies, like for example, like for example asking the teacher for help, or try to avoid those children and go somewhere on his own for 5 minutes to relax*.

#### 3.3.6 Repetitive movements/stereotypies

Three parents reported an improvement in repetitive movements and stereotypies during the clinical study. One of these parents described that the stereotypies totally disappeared during the clinical study.

*IT303: He was more relaxed to the point that stereotype disappeared completely. He is now doing it again but before the drug, he would do it a lot, even with his hands only, with his fingers in front of his eyes. Instead, with the drug bit by bit he stopped doing it. I was often so surprised about it. I used to say: “It’s impossible.” We did years and years of therapy, because he would play only with one cube, he would take everything at home and at school and he would stereotype it. He would self-stimulate himself a lot*.

#### 3.3.7 Eating

Two parents described an improvement in eating patterns. One parent described an improvement in the variety of food that the child started to accept while the other parent described an improvement in the ability of the child to feed himself.

*UK002: […], the positive… […] And eating-wise, now if… say, like, for example, if I cut the apple, if I leave it, leave it in the plate, he do eat by his self*.

*UK001: What else did I… He’s eaten, um, different foods. He’s eating more foods. He used to just eat, um, Coco Pops, apples, and waffles and it had to be a normal waffle, but now he’s eating fish fingers. He is now eating burgers. He did eat a little bit of sausage before, but now he’s burgers. He’s eating pizzas. I know this sounds like rubbish food, but we were eating… He was eating nothing, um, before, um, and it’s really, really, really, um…*

#### 3.3.8 Sleeping

Only one parent reported an improvement in sleeping issues with their child, although the issues did not completely disappear.

*UK002: Okay. Before, he used to wake up every two, three times a week in middle of the night. […] At least. But now, he sleeps like twice in two weeks’ time, he wakes up. There’s sometimes he doesn’t wake up, but he do wake up but not that much*.

#### 3.3.9 Resting state

Many parents (6 out of 11) described that their children were more calm/serene during the clinical study.

*IT303: As he started taking the drug, we immediately noticed he was more relaxed, and as he became more relaxed, he started listening to us when we would talk to him. He would pay attention to us when we would tell him to do something new, even the therapists noticed this aspect. He has 15 hours a week at home with therapists. The therapists as well noticed this obvious improvement. It has improved my life as well, because if he is calm, I’m calm as well. I can do many things. I was able to do many things more. If he is calm, and I’m not worried about him… for example, he would all of a sudden start crying, or start laughing, my day was devoted to him. The drug has changed my daily life as well, because I could go everywhere. I was feeling calm, I would call him and he would answer me, it was easier even spending time with him*.

*UK002: Because he learned quite a few things. Uh, he’s a bit calmer now*.

#### 3.3.10 Explorative relationship between caregiver’s experience and clinical data

Although this is a qualitative study, as there is a scarcity of data on autism, we explored the relationship between clinical outcomes and improvement reported by the parents. The results do not seem to show a relationship between parents’ reported improvement and clinical data. Indeed, three parents who reported the strongest improvement were in the control group. The improvement captured at week 26 with the different instruments in the clinical studies did not seem to capture the improvement in the burden described by the parents in our interviews. Indeed, only one of the three patients described by their parents as having improved in a major way had improved more than 3 points on the CARS-2 at week 26.

## 4 Discussion

The interviews produced rich qualitative accounts of the improvements noticed by parents during the clinical study. The purpose of the current qualitative study was to investigate the impact of bumetanide on the core symptoms of autism. The exit interview study provides an insight into autism. To the best of our knowledge, it is the second time that qualitative exit interviews were conducted with parents/caregivers of ASD patients enrolled in a phase 3 clinical study.

Patients could present a wide variety of different symptoms – the children were thus described by their parents on a broad spectrum of functioning. The findings show that parents consider some symptoms of ASD to be more bothersome than others. While communication and social skills and the inflexibility towards routines were most often described as being most bothersome and a great source of worry for the caregivers, other core symptoms such as repetitive behaviours and restricted interests were often seen as more manageable and least bothersome.

Caregivers in our study described that restricted interests and repetitive behaviours vary widely in type, frequency, and intensity among children and adolescents with ASD. Although they did not report these symptoms as most bothersome, they recognised that these behaviours could be stigmatising and interfere with social activities and the construction of social relationships. Therefore, the present results do not suggest that restricted interests and repetitive behaviours should be overlooked in further research but are consistent with the previous findings in the literature.

Aggressive behaviours in children with ASD are well documented in the ASD literature and often cause families a great deal of difficulty. It is not surprising that aggressive behaviours were reported as most bothersome by the caregivers whose children were displaying aggressive behaviours. Caregivers in this study described the stress and burden caused by self and other harm, which are common behaviours during a temper tantrum or meltdown, confirming that aggression is a major issue for caregivers of children with ASD. Future research may want to consider the need to address aggression in children with ASD.

An interesting finding in this study is the report by parents that they felt that their child was calmer and more relaxed. The sense of serenity at rest was described by the parents as life-changing experience for the parents as well as the children. The children were seen as more receptive of the requests of their environment and the parents in return reported feeling less tension and being able to relax themselves as well. These findings are in line with a small open-label study on six patients treated with bumetanide, where parents reported some developmental progress in their child based on the Parental Satisfaction Survey (PASS) (14), and a recent qualitative exploration in exit interviews of changes observed in clinical trials for individuals with ASD without intellectual disability (15).

This feeling of calm—or serenity, as the parents phrased it—is not captured by either the CARS-2 or the SRS, but merit to receive more attention in further clinical studies.

Although not all parents experienced a noticeable change in their daily life, all parents but two experienced improvements in their children’s behaviours during their participation in the clinical study. Parents of children described as having a lower deficit in communicative skills reported the least improvement. All those parents were hoping for an improvement in social skills as they saw a lack of social relationships of their children as one of the most bothersome symptoms. Improvements in social skills, the ability to engage in social activities, and show interest in others or build friendships did not seem to improve substantially. However, the development of social skills is a lengthy process. The length and the setting of a clinical study may not be suitable to capture improvements in newly acquired skills.

Nevertheless, the results show that meaningful improvements can be seen in children who remain severely autistic and that, in fact, any improvement is considered meaningful for parents.

*ES007: They certainly were, because any improvement, no matter how big it is, it’s positive*.

In our sample, we did not see a clear link between the severity of ASD as captured by the clinical data and parents’ reported burden and severity of symptoms. Parents described changes during the clinical study that were not or not well reflected by the clinical measures like the CARS-2 or the SRS. This could be explained by the fact that both scales focus on the core symptoms of ASD only. There is also a disconnect between the Clinician Global Impression of improvement and the parent impression of improvement. This could be explained by the fact that the measurements of clinical data were limited to the first 26 weeks of the clinical study, while parents reported the changes that they noticed during the whole duration of the clinical study. In addition, we asked parents to recall events over a 1-year period. It is possible that the length of the recall period may play a role in the discrepancies.

One possible explanation is that the perception of the parents was altered by the hope they were having when enrolling in the clinical study. Hope is affected by various factors, and it is defined as the likelihood of a better future rather than a hard and uncertain present. Previous studies have described the negative correlation between hope and burden, with caregivers reporting a lesser disease burden and a better quality of life when they had hope for improvements (16). In addition, our study was not formally designed to address this question and our sample is limited. Nevertheless, we believe it was interesting to report as this may impact future trials in autism.

In addition, the qualitative findings highlight the strong placebo effect experienced in the study. It is an interesting finding that all but one caregiver of the patients in the placebo group reported at least a minimal improvement, with some reporting a major improvement. The placebo effect is well documented in mental illness. It is possible that hope among families/caregivers is the foundation for a strong placebo-by-proxy effect in ASD, an effect that is independent from treatment expectations. As ASD symptoms are not likely to decrease because of spontaneous remission, there are several possible explanations for this effect. It is possible that the reduction in symptoms is due to the mere act of participating in a research study. Another explanation would be that caregivers behaved differently during the course of the study, displaying placebo-by-proxy behaviours that in turn impacted the behaviour of their child. Caregiver ratings seemed to be more prone to a placebo-by-proxy response in social communication difficulties. It has been argued that placebo-by-proxy effects are important components of a placebo response in child/adolescent psychiatry, since they can alter caregivers’ perceptions of symptoms and/or modify caregivers’ behaviours towards children and subsequently improve symptoms (thus improving scores also in non-caregiver scales). Placebo responses represent a major challenge in psychopharmacological drug development in ASD, where large clinical trials were unable to demonstrate efficacy over placebo, despite robust preclinical findings. For this reason, key factors that could affect placebo responses, including the type of outcome measures to include, should be considered when designing such clinical studies. This is especially the case for studies where subjective measures of improvement are used to detect differences between placebo and the active treatment.

## 5 Limitations

The findings in this study were based on caregivers’ reports in a timeframe different from the timeframe to collect improvement in the clinical studies with standardised measures. As such, inaccurate reporting might have biased our findings. Recall bias may have impacted the findings, as the caregivers were asked to recall improvements and the extent of the improvement over a period of 1 year.

The sample size is small, with only 11 caregivers included in the study. Although saturation was reached in terms of the symptom domains, the sample size is limited.

Although the participants were asked to describe the impact of the treatment, both positive and negative, no prompts were included to actively probe into potential adverse events of the treatment. Adverse event reports were therefore only spontaneous and may be underrepresented in our sample.

Despite these limitations, this study provided a valuable picture of the burden of ASD and provides valuable insights into the improvements the parents have experienced during the clinical study and highlighted the need for future research to better understand the relationship between clinical outcomes and patients’ and caregivers’ experiences.

## 6 Conclusion

Parent and caregiver perspectives on the effectiveness of therapies available to their children are important but neglected in the clinical studies of patients with ASD. In recent years, there has been increasing recognition of the value of caregiver perspectives for informing the assessment of treatment benefit in clinical settings. Exit interviews conducted after completion of clinical trials provided a rich source of qualitative data, allowing a detailed understanding of the parents’ and patients’ experiences of ASD and of what constitutes a meaningful change in ASD symptoms.

These data also helped identify important experiences that may enable the development of more patient-centric clinical trials, e.g., with novel measurement strategies evaluating meaningful changes in ASD outcomes. Indeed, parents described a sense of serenity that is not currently captured by COAs. We believe this innovative approach could benefit other clinical trials, ultimately helping to address patient and caregiver needs.

## Data Availability

The raw data supporting the conclusions of this article will be made available by the authors, without undue reservation.
